# An open letter to the NIH: value and costs in publishing

**DOI:** 10.1172/JCI200201

**Published:** 2025-10-01

**Authors:** Sarah Jackson, Karen D. Guth, John B. Hawley, Corinne L. Williams, Priscilla Y. Hsue, Oliver Eickelberg, Elizabeth M. McNally

**Affiliations:** 1 Executive Editor, *The Journal of Clinical Investigation and JCI Insight*.; 2 Managing Director, The American Society for Clinical Investigation.; 3 Executive Director, The American Society for Clinical Investigation.; 4 Senior Science Editor, *JCI Insight*.; 5 President, The American Society for Clinical Investigation.; 6 Editor in Chief, *JCI Insight*.; 7 Editor in Chief, *The Journal of Clinical Investigation*.

*We share here our response to the NIH’s request for information on maximizing research funds by limiting allowable publishing costs.*

Dear Dr. Bhattacharya and Colleagues at the NIH,

The American Society for Clinical Investigation (ASCI) self-publishes 2 highly rigorous journals in the biomedical research landscape, *The Journal of Clinical Investigation* (*JCI*) and *JCI Insight*. The ASCI is a nonprofit honor society of physician-scientists representing all medical specialties. The *JCI*, which began publishing in 1924, is ranked fourth among 190 journals indexed in the “Medicine, Research & Experimental” category of *Journal Citation Reports* and is among the most prestigious and reputable journals in biomedical science. All of the *JCI*’s research content has been freely available since the journal first appeared online in 1996; *JCI Insight* has been freely available since its inception in 2016. Importantly, the *JCI* and *JCI Insight* are unique as self-published journals that are not reliant on large publishing houses. The ASCI employs a small, entirely US-based staff to handle all aspects of the publishing process — from initial receipt of a manuscript to its final publication. This structure makes us particularly well suited to comment on the true costs of publishing a highly rigorous US-based scientific journal. The *JCI* and *JCI Insight* have been leaders in scientific rigor and integrity in publishing; hence, we are also well-positioned to comment on the journal’s role in ensuring the rigor and reliability of published science.

## Recommendation for allowable publishing costs

**Figure 1:**
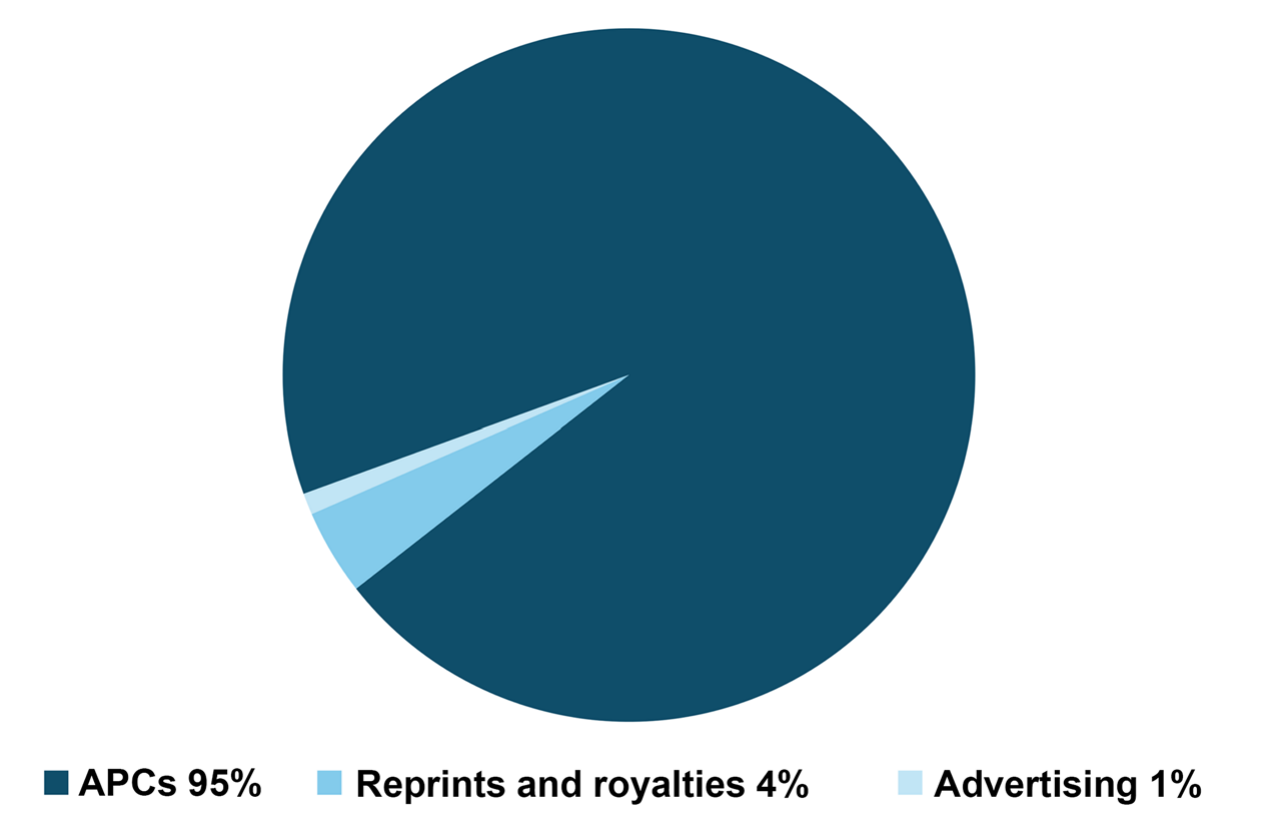
Sources of Journal Revenue. The *JCI* and *JCI Insight* primarily receive revenue through article processing charges (APCs).

We applaud the NIH’s efforts to put checks and balances in place in regard to publication costs. We agree that select for-profit journals have very high article processing charges (APCs), in addition to revenues derived from institutional and individual subscriptions. The *JCI* and *JCI Insight* are gold open-access journals almost entirely funded by APCs (Figure 1). Our journals have no subscription revenue and minimal royalty and advertisement revenue. The APCs that we charge cover the costs of publication (Table 1), with an average of just 3% of APCs going toward support of ASCI programs in the past five years. Publishing does not produce a windfall for the society; rather, the journals serve as one mechanism to fulfill the ASCI’s mission to support the scientific efforts, educational needs, and clinical aspirations of physician-scientists to improve the health of all people.

We suggest that the NIH focus its policies on mandating that NIH-supported studies be published in fully open-access journals, rather than hybrid journals that charge both high APCs and subscription fees. We trust that the *JCI*/*JCI Insight* model demonstrates the viability of the gold open-access model in a rigorous and competitive scientific publishing landscape. We agree that it is reasonable to request a limit on allowable costs per publication (listed as Option 2 in the NIH “Request for Information on Maximizing Research Funds”). Based on our nearly 30-year history of providing free access to research, *we recommend that the limit on allowable costs per publication be set at $6,000, with annual adjustments for inflation*. This price point is based on our experience as a US-based, nonprofit organization that spans all steps in the evaluation and publication process, including editorial boards and staff; copy editing and layout of published content; IT staff responsible for manuscript submission and publishing platforms; and downstream delivery of our content to PubMed, PubMed Central, Crossref, the Directory of Open Access Journals (DOAJ), and other content repositories.

## Relevant comparators

A recent NIH analysis used publicly available data from the DOAJ to gauge the range of publication costs. This use of DOAJ as a reference point for allowable APCs, however, fails to appropriately consider costs associated with journals that publish both preclinical and clinical research studies. There are higher costs associated with evaluating the in-depth experimental work beyond what is incurred by journals publishing in other disciplines. It is important when comparing journals to consider both the scope and the quality of the content published. We have appended a table of open access APCs in journals publishing high-quality biomedical research (Supplemental Table 1; supplemental material available online with this article; https://doi.org/10.1172/JCI200201DS1). The table delineates full open access journals and those with a hybrid model, providing a relevant comparator dataset for APCs. We recommend that the NIH consider APCs from open access journals that currently or historically have published NIH-funded studies (i.e., the average cost per publication specifically in open access journals, which are meeting goals of public access).

## What is the value of the publication process?

To assess submitted content, the *JCI* and *JCI Insight* rely primarily on academic editors who are active researchers themselves. With broad scientific knowledge, our editors scrutinize the rigor, quality, and potential impact of manuscripts both before and after the peer review process, relying on their ongoing experiences as active investigators. At the *JCI*, about 25% of submitted articles are judged to be of a quality and impact deserving of more detailed external peer review. For this, we depend on input provided by subject matter experts who provide technical analysis of the research under consideration, as well as assessment of the work’s importance, as part of the peer review processes (see, *Should we pay for peer review?*). Nearly all manuscripts ultimately accepted undergo one major revision, necessitating an additional round of editor/reviewer assessment before acceptance. For the top 10% of papers that we determine to be acceptable in principle, we then apply another round of rigorous screening that utilizes both software tools and expert professional staff to identify potential issues with statistical analysis, data presentation, provision of raw data, appropriate database deposition, and, critically, data integrity. This last step requires extensive human effort, aided by software, and is essential for reproducibility and reliability.


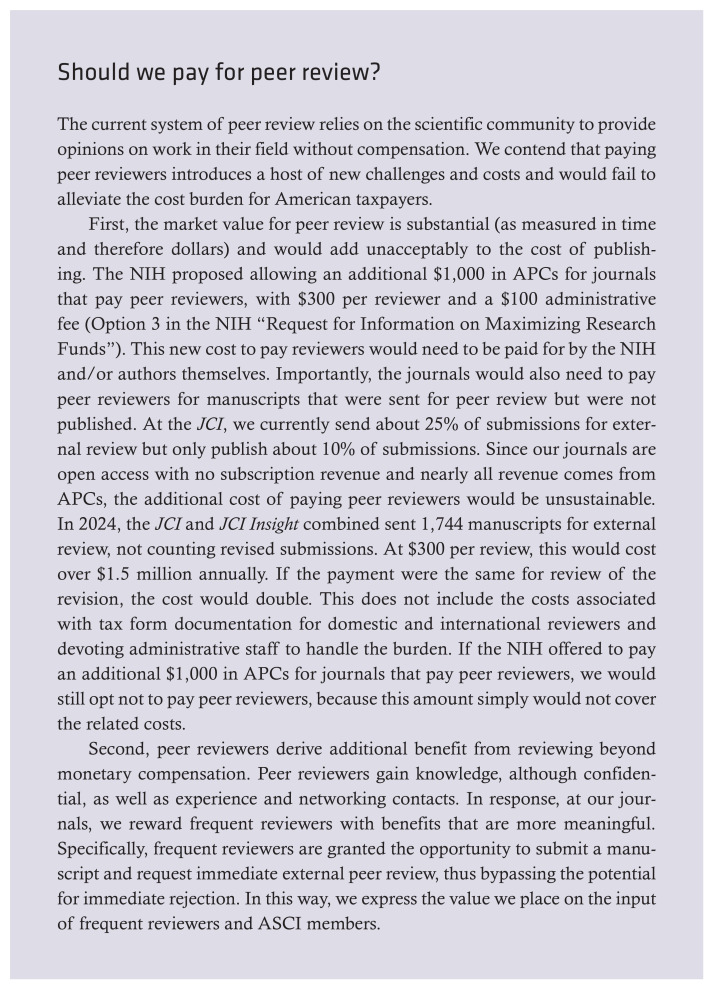


While software for artificial intelligence–based (AI-based) image screening and plagiarism screening plays a crucial role in this process, these tools are insufficient on their own. The output of any screening software still requires staff members and editors to interpret the findings, as those screenings are known to produce false-positive as well as false-negative results. Humans still need to understand the context of any identified issues (e.g., there could be acceptable reasons for similar images in a manuscript, including time-course experiments, serial sections for histology, single and multichannel images of the same immunostained sample). In cases with uncertainty about any issue, we devote extensive time to asking authors for an explanation of potential irregularities for evaluation by the editors. Ensuring adherence to standards of transparency and reliability involves substantial staff efforts that are not covered by any available software, such as manually screening data for transparent presentation (e.g., whether the paper clearly shows the number of replicates in graphs and provides supporting values underlying graphical data), ensuring that high-throughput data are deposited in publicly accessible repositories, manually vetting raw data for Western blot experiments, and assessing statistical analysis. For clinical studies, staff members also cross-check trial registration, ensure that the ClinicalTrials.gov has been appropriately updated, vet conflict-of-interest disclosures, and evaluate clinical trial checklists. The *JCI* and *JCI Insight* are among the most rigorous in taking these added measures beyond the peer review process. Importantly, we view these steps as vital to ensuring that the content we publish is of the highest caliber.

Importantly, recent advances in AI have allowed incredible advances in scientific discovery. At the same time, however, the scientific community faces growing threats from fraudulent papers (generated in well-described paper mills), entailing added time for expert review, as described above. In recent months, more and more scientific submissions and peer reviewer comments have been documented to be completely generated by AI. In a time when anyone can post anything online, it is more important than ever that journals assess scientific findings at all levels for veracity and integrity, verify the identity of peer reviewers, and ensure trusted content. We emphasize the importance of this validation process for journals that publish laboratory-conducted experiments, which have data that can and should be made available for all readers to evaluate, consistent with NIH policies.

Publishers also play a key part in helping meet NIH goals for transparency in data reporting and data availability. At the journal level, we ensure that published articles include a relevant statement on data availability and enforce requirements to deposit data in public repositories, whenever those repositories exist. The journals also host raw manuscript data directly on our website. This includes gel and immunoblot images and individual data values underlying presented graphs. Every single one of these steps has associated costs.

## How low is too low?

Our concern is that pushing for the lowest minimum APCs would compromise the ability of journals to ensure the quality of science that is published and directly impact publication of reputable, rigorous, and reproducible science. The journals play a key role in vetting the quality of science through their processes of editorial evaluation, reevaluation, peer review, and data integrity scans.

In addition, low allowable APCs favor the large for-profit journals that publish at scale and would hence have the opposite intended impact. The economy of large for-profit journals enables them to more readily trim margins, including outsourcing publishing work to overseas vendors and reducing or eliminating employment opportunities in the US. It is critical that any change in NIH publication policy does not disadvantage small, independently published journals. Journals hosted by academic societies have an important role in publishing science of the highest quality, because these are the journals with the greatest input from active scientists. The ASCI is particularly well poised to evaluate quality and rigor in research and deliver content across the translational medicine spectrum. This translational spectrum is a pain point for drug development, and therefore it is critically important to have independent, high-quality scientific assessment. There are many other examples of academic societies representing the top researchers within a given field that are equally well positioned to serve as stewards of the scientific literature. The NIH’s effort to curb costs should not come at the expense of sacrificing content quality provided by nonprofit publishers. We are concerned this effort could adversely affect or even eliminate academic society journals that enable top researchers in the field to be involved as both editors and peer reviewers. While academic society journals make up a small percentage of journals, the research community should not cede all benefit from having choices beyond the Nature/Springer and Cell Press/Elsevier portfolios.

We also note that restricting allowable APCs favors larger research entities that may have access to additional, non-NIH funds to cover costs over the allowable APCs. This disadvantages smaller, less research-intensive institutions.

## Concluding remarks

As a nonprofit publisher, we are faced with increasing demands in evaluating the quality of work at the same time as cost restrictions from funders. The NIH as a funder should have a role in both avoiding excess costs and ensuring that funded science is rigorously evaluated in the publishing process. We believe that setting a limit on allowable costs per publication at $6,000 and mandating publication in an open-access journal will balance cost, quality, and access. The best value for the American taxpayer is to ensure that NIH-funded APCs yield high-quality, accessible content at a reasonable cost without impeding free discussion of academic findings.

## Supplementary Material

Supplemental table 1

## Figures and Tables

**Table 1:**
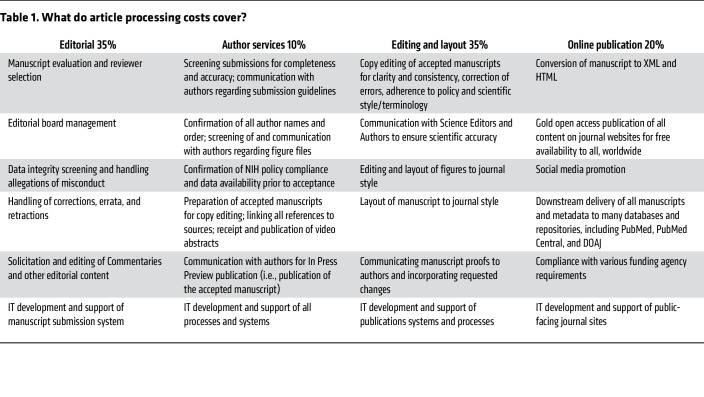
What do article processing costs cover?

## Version 1 09/25/2025

Electronic publication

