# Supplementary material for: An open letter to the NIH: value and costs in publishing
Source: J Clin Invest. 2025 Oct 1;135(19):e200201. doi: 10.1172/JCI200201 (PMC12487954; doi:10.1172/JCI200201)
Supplement: Supplemental table 1 [file jci-135-200201-s319.pdf]

Open Access (OA) Article Processing Charges (APCs)

Open Access Journals	APC for OA	Source
EMBO Molecular Medicine*	$7,990	Author Guidelines | EMBO Molecular Medicine
Nature Communications	$6,990	Open Access Fees and Funding | Nature Communications
PLOS Medicine*	$6,460	Explore our publication fees and funding for open access publishing PLOS
eBioMedicine	$6,360	Open Access | The Lancet
Journal of Clinical Investigation*	$5,700	JCI - Author Information Center
Cell Reports	$5,620	Open access | Cell Press
Science Advances*	$5,450	Licensing and charges | Science | AAAS
JCI Insight*	$4,900	JCI Insight - Author Information Center
Blood Advances*	$4,000	Publication Fees | Blood Advances | American Society of Hematology
Journal of Biological Chemistry*	$3,430	Open Access | Journal of Biological Chemistry
Scientific Reports	$2,690	Open Access Fees and Funding | Scientific Reports
**Subscription Journals with Open Access option**	**APC for OA**	**Source**
Nature Medicine	$12,690	Publishing options | Nature Medicine
Cell	$11,400	Open access | Cell Press
Cell Metabolism	$10,400	Open access | Cell Press
Cancer Research*	$8,000	Publication Fees and Reprints | American Association for Cancer Research
Blood*	$6,600	Publication Fees | Blood | American Society of Hematology
Journal of Neuroscience*	$6,270	Information for Authors | Journal of Neuroscience
Journal of Experimental Medicine*	$6,000	Submission Guidelines | Journal of Experimental Medicine | Rockefeller University Press
Proceedings of the National Academy of Sciences*	$5,495	Publication Charges | PNAS Author Center
Oncogene	$5,490	Open Access & Self Archiving | Oncogene
American Journal of Respiratory and Critical Care Medicine*	$5,475	AJRCCM Instructions for Contributors ATS Journals
Journal of the American Society of Nephrology*	$5,250	Article Charges | ASN Publications
Circulation*	$5,400	Author Hub | AHA/ASA Journals
Gastroenterology	$4,180	Open Access Policies | Gastroenterology | AGA
Journal of Immunology*	$3,710	Charges, licences, and self-archiving | Oxford Academic

* Non-profit journal
